# Commentary: Association between systemic immune-inflammation index and psoriasis: a population-based study

**DOI:** 10.3389/fimmu.2024.1425182

**Published:** 2024-06-13

**Authors:** Xiao Li, Yonglong Hao, Meirong Chen

**Affiliations:** ^1^ First Clinical Medical College, Shandong University of Traditional Chinese Medicine, Jinan, China; ^2^ The Second Affiliated Hospital of Shandong University of Traditional Chinese Medicine, Jinan, China; ^3^ Department of Ophthalmology, Shandong Hospital of Traditional Chinese Medicine, Jinan, China

**Keywords:** SII, psoriasis, Systemic Immune-Inflammation Index, National Health and Nutrition Examination Survey (NHANES), cross-sectional studies

## Introduction

We read with interest the article entitled “Association between systemic immune-inflammation index and psoriasis: A population-based study” by Zhao et al. This study utilized data from the National Health and Nutrition Examination Survey (NHANES) from 2009 to 2014 to innovatively explore the association between the Systemic Immune Inflammatory Index (SII) and psoriasis in the US population. Multivariate linear regression modeling revealed a significant positive association between SII and psoriasis, and a subsequent sensitivity analysis, by converting SII from a continuous to a categorical variable, found that each one-unit increase in SII increased the risk of psoriasis for participants in the highest quartile compared with those in the lowest quartile by 64.5% (OR = 1.645; 95% CI, 1.261 –2.145, *p* < 0.05). In addition, the authors conducted subgroup analyses to explore the association between SII and psoriasis in different populations based on gender, body mass index, stroke status, and cancer status. This is a very innovative and interesting study, but we have some questions about the study.

## Statistical methods

The authors mention in the paper that SII was designated as the exposure variable and psoriasis status as the outcome variable, yet it is mentioned in the statistical analysis section that the beta value and 95% confidence interval are generated through multivariate logistic regression, thus whether it should be an OR value. Moreover, when converting SII to categorical variables. The sensitivity analysis shows that SII was converted to quartiles, but the abstract section shows that SII was converted to tertiles. We believe that this error should be avoided to minimize the impact on the reader.

## Covariate selection

The authors adjusted for important covariates such as age, gender, and race in their study, which is very commendable. However, due to the prolonged course of psoriasis and its heavy financial burden (1–3), we would highly encourage the authors to include the poverty index as an important covariate in this study to obtain more accurate findings. They can also elaborate on the definition of covariates such as smoking, alcohol consumption, etc., and whether this was done through questionnaires or specialized testing tools.

## Assessment of predictive capacity

The SII is a novel indicator for assessing systemic immune inflammation based on calculations of neutrophil, lymphocyte, and platelet counts. The authors also note that SII may be a useful tool for monitoring psoriasis activity and treatment efficacy. However, the authors did not assess the predictive ability of the SII based on the receiver operating characteristic. We also encourage the authors to compare the predictive ability of the SII with the neutrophil-to-lymphocyte ratio, platelet-to-lymphocyte ratio, and monocyte-to-lymphocyte ratio, as this would help us to assess whether the SII could be more widely used as a better predictive tool in the future.

## Discussion

Overall, this is an interesting study, but we hope that the authors will take care of our questions and make the article more accurate and complete so that readers will have a better understanding of the relationship between immunoinflammation and psoriasis.

## Author contributions

XL: Writing – original draft. YH: Writing – review & editing. MC: Writing – review & editing.

## References

[1] Zhao X Li J Li X. Association between systemic immune-inflammation index and psoriasis: a population-based study. Front Immunol. (2024) 15:1305701. doi: 10.3389/fimmu.2024.1305701 38504983 PMC10948528

[2] Jung S Lee SM Suh D Shin HT Suh DC. The association of socioeconomic and clinical characteristics with health-related quality of life in patients with psoriasis: a cross-sectional study. Health Qual Life outcomes. (2018) 16:180. doi: 10.1186/s12955-018-1007-7 30208968 PMC6136229

[3] Eley SJ DeMeo DP Korman NJ Carroll BT. Equity in the usage of biologics for psoriasis in the working poor. Arch Dermatol Res. (2023) 315:1029–31. doi: 10.1007/s00403-022-02410-7 36307556

